# Flexible mini-percutaneous nephrolithotomy versus retrograde intra-renal surgery in the management of renal stones: a randomized controlled trial

**DOI:** 10.1007/s00345-025-05689-z

**Published:** 2025-05-26

**Authors:** Ahmed Higazy, M. kandil, Ahmed Elshafei, M. Esmat, A. M. Tawfeek

**Affiliations:** 1 Urology Department, Faculty of Medicine, Ain Shams University, Cairo, Egypt; 2 Urology Department, Faculty of Medicine, Modern University for Technology and Information, Cairo, Egypt

**Keywords:** Cystonephroscope, Flexible mini-percutaneous nephrolithotomy, Mini-percutaneous nephrolithotomy, Retrograde intrarenal surgery, Stones

## Abstract

**Purpose:**

To assess the safety, efficacy, and cost-effectiveness of flexible mini-percutaneous nephrolithotomy (F-mPCNL) in managing nephrolithiasis compared to retrograde intrarenal surgery (RIRS).

**Methods:**

130 adult patients with renal stones (1.5–3 cm) were randomized to F-mPCNL or RIRS. The stone-free rate (SFR) was our primary outcome. Secondary outcomes were the operative time, fluoroscopy time, DJ stent insertion rate, auxiliary procedure needed, hospital stay, complications rate, total cost, and quality of life for both procedures.

**Results:**

out of 130 patients in our study, 125 were evaluated and completed the follow-up period. Preoperative data was similar in both groups including stone characteristics. F-mPCNL showed a better SFR of 95.1% compared to 77.8% in the RIRS group (p-value˂0.001), this was associated with less need for additional procedures. F-mPCNL was associated with a shorter operation time of 47.60 ± 14.54 min compared to 59.30 ± 20.10 min in the RIRS group (p-value˂0.001). F-mPCNL showed less radiational exposure and less need for DJ stent insertion than RIRS. The overall complication rate was comparable in both groups, postoperative pain, need for extra doses of analgesics, and hospital stay were higher in the F-mPCNL group. In our study, F-mPCNL was shown to be more cost-effective than RIRS.

**Conclusion:**

F-mPCNL showed a superior SFR in treating renal stones compared to RIRS in a single session, with less need for auxiliary procedures, shorter operative time, less radiational exposure, and better quality of life. However, it was associated with slightly more hospital stay and postoperative pain compared to RIRS.

## Introduction

Retrograde Intrarenal Surgery (RIRS) is an effective minimally invasive treatment for renal stones. Compared to percutaneous nephrolithotomy (PCNL), RIRS offers the benefits of less perioperative morbidity, less risk of bleeding, no risk of bowel injury, and a more favorable recovery process [1–4].

RIRS has some disadvantages, such as the lower efficacy of stone clearance compared to PCNL, which is directly proportional to the stone size. This raises the need for a DJ stent to allow the passage of fragments smoothly leading to repeated interventions, exposing the patient to lower urinary tract symptoms (LUTS), and affecting their quality of life (QoL) [5].

Minimally invasive percutaneous nephrolithotomy (mPCNL) has emerged as an effective, and less invasive treatment option for removing large renal stones compared to conventional PCNL. However, the rigidity of such nephroscopes limits maneuvering into renal calyces at acute angles. This limitation may require additional percutaneous tracts, leading to increased morbidity [5–7].

The recent introduction of a single-use flexible mini-percutaneous nephrolithotomy (F-mPCNL) through a medium-sized tract using a flexible cysto-nephroscopy device aimed to tackle previous challenges. It allows access to all regions of the pelvi-calyceal system through a single small percutaneous access tract to combine the benefits of PCNL and RIRS [8, 9].

This has prompted us to evaluate F-mPCNL using flexible single-use cysto-nephroscopy compared to RIRS in managing renal stones regarding stone-free rate, perioperative morbidities, patients’ quality of life, and cost-effectiveness.

## Methods

The study was held between January 2024 and October 2024. Patients above 16 years of both sexes with renal stones size between 1.5 and 3 cm in greatest dimension as determined by non-contrast computed tomography (NCCT) were included in our study. Patients with lumber hernia on the same side of the stone, stone burden exceeding 3 cm, renal anomalies preventing renal access, coagulopathies/bleeding tendencies, or urinary tract infections were excluded.

Our patients were randomized and sorted into two equal groups with a 1:1 ratio after obtaining informed consent using a computer-based allocation for the F-mPCNL and RIRS surgical groups. The principal investigators, the data collector, and the statistician were blinded to the type of intervention until the procedures were selected randomly.

The stone size was determined using the greatest dimension in NCCT. The stone volume (burden) was evaluated using the formula: Total Stone Volume (TSV = stone width x stone length x stone depth x π x 0.167) on non-contrast computed tomography [6, 10].

### Surgical technique: flexible endoscopic mini-percutaneous nephrolithotomy

Under general anesthesia, patients were positioned in the Galdakao-Modified supine Valdivia position and the renal puncture was done by fluoroscopic guidance medial to the posterior axillary line using an 18-gauge puncture needle [11, 12].

Tract dilatation was done with Storz 15/16 Fr one-step metal dilator followed by a 16.5 Fr access sheath. We utilized the flexible endoscope (WiScope^®^ Single-Use Digital Flexible Cystoscope OTU-C380RR, Medical, California, USA). Laser stone lithotripsy was performed using 550 μm fiber (Maxi Fibre, NEUWEG Medizintechnik, Hallbergmoos, Germany) and device setting (SHPINX^®^ JR Holmium laser device, Germany- Power: 25-W, 2.5 J, 10 Hz frequency).

A Dormia basket/stone forceps were used to extract fragments in addition to saline irrigation. Occasionally, a clamped nephrostomy tube was placed for 24 h based on the surgeon’s decision when it was needed. The D.J. stent was left in place for 2–4 weeks in case of hematuria or significant stone fragments at the end of the operation; otherwise, a ureteral catheter was left in place. The nephrostomy tube and the ureteral catheter were removed on the first day postoperatively.

### Retrograde intrarenal surgery

RIRS was performed in the lithotomy position. Serial ureteric dilatation was done, and an 11/13 Fr access sheath was inserted under fluoroscopic guidance. LithoVue™ single-use Digital Flexible Ureteroscope (Boston Scientific, Massachusetts, U.S.) was used in all patients.

Laser stone dusting was done using 272 μm fiber, device setting (power: 17-W, 0.7 J, 10 Hz frequency). At the end of the procedure, we routinely placed a postoperative DJ stent for 4 weeks.

Operative time, fluoroscopy time, and perioperative complications were recorded. On the same day of the procedure, a visual analog (VA) pain scale was used to evaluate postoperative pain. Non-steroidal anti-inflammatory (NSAID) medication was prescribed for postoperative analgesia; in case of a narcotic requirement, it was given upon request and documented.

All patients underwent NCCT one month postoperatively to detect any residual stone ≥ 4 mm. The need for auxiliary procedures for significant stone residuals, including ESWL sessions, redo endoscopy, or chemo-dissolution, was recorded. Quality of life was assessed in our study population after one month using the SF-36 questionnaire [13].

Our primary outcome was to assess the SFR of F-mPCNL and RIRS one month after surgery. Secondary outcomes were to evaluate the operative time, fluoroscopy time, need for DJ Stent, hospital stay, postoperative analgesia, complications rate using the Clavien-Dindo classification, need for auxiliary procedures, and the cost-effectiveness of both procedures.

The cost analysis was done at 2024 rates. The cost was calculated in our local currency and was converted into USD for standardization. Procedural costs included operating room use, the mean cost of the instruments, dilators, DJ stent, fluoroscopy time, hospitalization, the cost of auxiliary procedures, and management of complications if needed in addition to the cost of stent removal.

### Statistical analysis

Data evaluation was done using the SPSS 23.0 package program. Description of the quantitative variable was done as mean and standard deviation and student t-test was used to compare the two groups in parametric data while the qualitative data were compared using the Chi-square test. The confidence interval applied was 95% and the margin of error accepted was set to 5%. The p-value was considered significant as the following: P-value > 0.05: non-significant, P-value < 0.05: significant, and P-value < 0.01: highly significant.

## Results

**Fig. 1:**
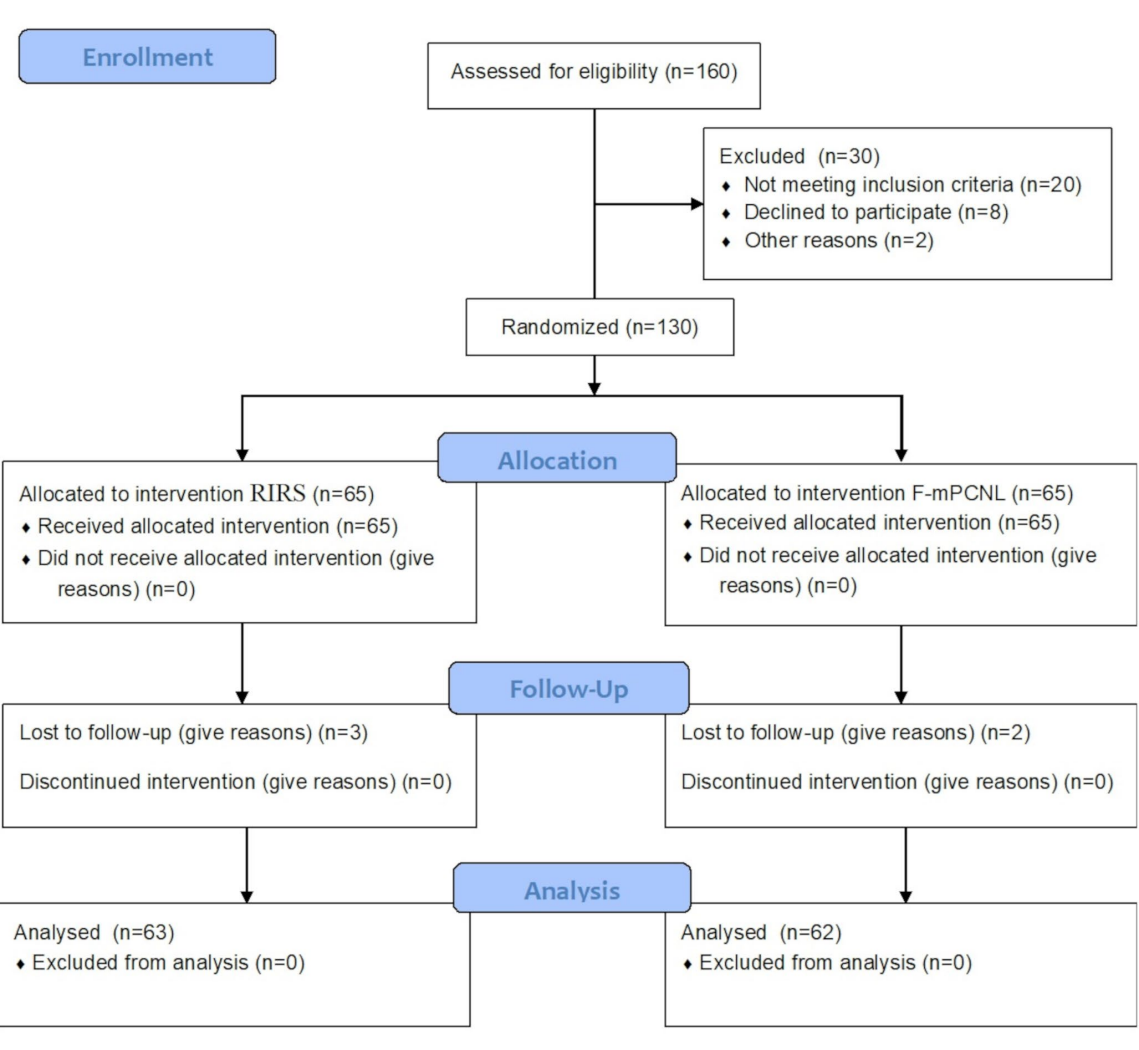
Consort flow diagram

Out of 130 patients in our study, 125 were followed up after 1 month and included in our assessment, as shown in (Fig. 1). Demographic data and preoperative stone parameters were similar in both groups, as shown in (Table 1).

**Table 1:** Demographic data and stone parameters

		RIRS *N* = 63	F-mPCNL *N* = 62	*P*-value
Age (years)		36.13 ± 11.38	39.89 ± 10.86	0.145
Sex	Females	18 (28.6%)	15 (24.2%)	0.408
	Males	45 (71.4%)	47 (75.8%)	
BMI		31.01 ± 6.31	28.41 ± 7.88	0.118
Previous renal intervention	Non	49 (77.8%)	47 (75.8%)	0.707
	ESWL	1 (1.6%)	3 (4.8%)	
	Pyelolithotomy	2 (3.2%)	1 (1.6%)	
	PCNL	6 (9.5%)	8 (12.9%)	
	RIRS	5 (7.9%)	3 (4.8%)	
Stone Size in maximum diameter (Cm)		2.42 ± 0.54 Range: 1.2-3	2.31 ± 0.49 Range: 1.1-3	0.965
Total Stone Volume (Cm3) (stone width x length x depth x π x 0.167)		2.67 ± 1.44 Range: 1.57–2.72	2.78 ± 1.33 Range: 1.47- 3.0	0.644
Stone laterality	Right	34 (54.0%)	29 (46.8%)	0.359
	Left	29 (46.0%)	33 (53.2%)	
Stone Number	One	35 (55.6%)	38 (61.3%)	0.389
	Two	20 (31.7%)	19 (30.6%)	
	Three or more	8 (12.7%)	5 (8.1%)	
Stone Density (HU)		1122.5 ± 204.5	1045.5 ± 266.17	0.242
Stone Location	Pelvis	13 (20.6%)	8 (12.9%)	0.636
	Lower Calyx	8 (12.7%)	8 (12.9%)	
	Middle Calyx	5 (7.9%)	5 (8.1%)	
	Upper Calyx	2 (3.2%)	1 (1.6%)	
	Combined Pelvic and calyceal	25 (39.7%)	23 (37.1%)	
	Involving 2 or more calyx	10 (15.9%)	17 (27.4%)	

The perioperative data is shown in (Table 2). The mean operative time in the F-mPCNL was 47.60 ± 14.54 min compared to 59.30 ± 20.10 min in the RIRS with a highly significant difference (p-value ˂0.001). F-mPCNL was associated with less fluoroscopy time compared to RIRS. The need for DJ insertion in the F-mPCNL group was reported in 9 patients representing 14.5% of the whole group. A nephrostomy tube was inserted in 5 patients only (8.1%). 2 patients in the F-mPCNL group required a percutaneous puncture due to limited deflection with a steeply angled infundibulum after percutaneous entry with no reported added complications.

**Table 2:** Comparison between the RIRS group and F-mPCNL groups regarding perioperative data

	RIRS *N* = 63	F-mPCNL *N* = 62	*P*-value
Operative time (min.)	59.30 ± 20.10	47.60 ± 14.54	˂0.001
Fluoroscopy time (Sec)	68.51 ± 18.10	46.14 ± 6.35	˂0.001
DJ stent insertion	63 (100.0%)	9 (14.5%)	˂0.001
Nephrostomy tube application	-	5 (8.1%)	
Need for secondary puncture	-	2 (3.2%)	
Hb Drop	0.1 ± 0.2	0.31 ± 0.48	0.151
VA Pain scale	4.31 ± 1.05	5.73 ± 1.41	< 0.001
Hospital stay (days)	1.07 ± 0.34	1.89 ± 0.93	0.021

SFR was seen higher in the F-mPCNL with 95.1% compared to 77.8% in the RIRS group with a highly significant difference (p-value ˂0.001) as shown in (Table 3). For residual stone management, 5 patients in the RIRS and 1 patient in the F-mPCNL refused further intervention. 6 out of 14 patients in the RIRS underwent a single ESWL session compared to 1 patient in the F-mPCNL, and 1 patient underwent redo RIRS surgery in the RIRS.

**Table 3:** Comparison between the RIRS group and F-mPCNL group regarding outcome and postoperative data

		RIRS *N* = 63	F-mPCNL *N* = 62	*P*-value
Stone free rate		49 (77.8%)	59 (95.1%)	< 0.001
Residual stones > 4 mm		14 (22.2%)	3 (4.8%)	0.012
No intervention for residual stones		5	1	
Further management of residual stones	Total	9 (14.3%)	3 (4.8%)	0.043
	ESWL	6 (9.5%)	1 (1.6%)	
	Redo-surgery (RIRS)	1 (1.5%)	-	
	Chemo dissolution	2 (3.2%)	1 (1.6%)	
Postoperative complications (Clavien Dindo)	Overall	16 (25.4%)	23 (37.1%)	
	Grade 1 (pain)	10 (15.9%)	20 (32.2%)	< 0.001
	Grade 2 (fever)	6 (9.5%)	3 (4.8%)	0.310
Comprehensive Complication Index		11.75 ± 5.4	9.6 ± 3.3	0.047
Quality-Of-Life (sf-36)		74.43 ± 14.52	84.26 ± 11.43	< 0.001

Postoperative pain and requirements for extra doses of analgesia were seen higher in the F-mPCNL with no other significant difference in the peri-operative complication rate. We did not encounter any case with organ injury, urine leak, need for blood transfusion or grade 3,4, or 5 Clavien-Dindo classification.

Cost evaluation in our study is shown in (Table 4), the average total cost was less in the F-mPCNL compared to the RIRS groups with a highly significant difference (p-value ˂0.001).

**Table 4:** Cost evaluation for both procedures

	RIRS *N* = 63	F-mPCNL *N* = 62	*P*-value
Procedure cost (irrigation fluid, scopes, dilators,…)	1013.56 ± 98.21	697.53 ± 57.38	< 0.001
Hospital stay Cost	71.38 ± 20.87	115.74 ± 13.81	< 0.001
Auxiliary procedure Cost	392.00 ± 443.71	158.50 ± 72.83	0.498
Total cost (USD$)	1136.52 ± 223.70	818.10 ± 70.05	< 0.001

## Discussion

Nowadays, surgeons and patients prefer surgeries per natural orifices like RIRS with fewer co-morbidities, better stone handling, and pelvicalyceal navigation. On the other hand, PCNL provides better stone clearance, especially for those with a larger stone burden. Recently, mPCNL using a smaller caliber sheath was used to reduce PCNL morbidity with less renal parenchyma injury and less bleeding [5].

To overcome the disadvantage of the semi-rigid nephroscope in mPCNL, we used flexible single-use cysto-nephroscopy through percutaneous access to perform F-mPCNL, to allow better stone clearance and intrarenal navigation. This differs from UMPCNL which has a smaller access sheath limited to 11–13 Fr with a specifically designed instrument [14]. This small size limits the ability to address a large stone and SFR.

In our study, The stone size was 2.42 ± 0.54 cm and 2.31 ± 0.49 cm in the RIRS and F-mPCNL groups respectively. Both techniques were effective in the treatment of renal stones. However, F-mPCNL showed a higher single-session SFR of 95.1% compared to 77.8% in the RIRS group with a highly statistically significant difference (p-value < 0.001).

**Fig. 2:**
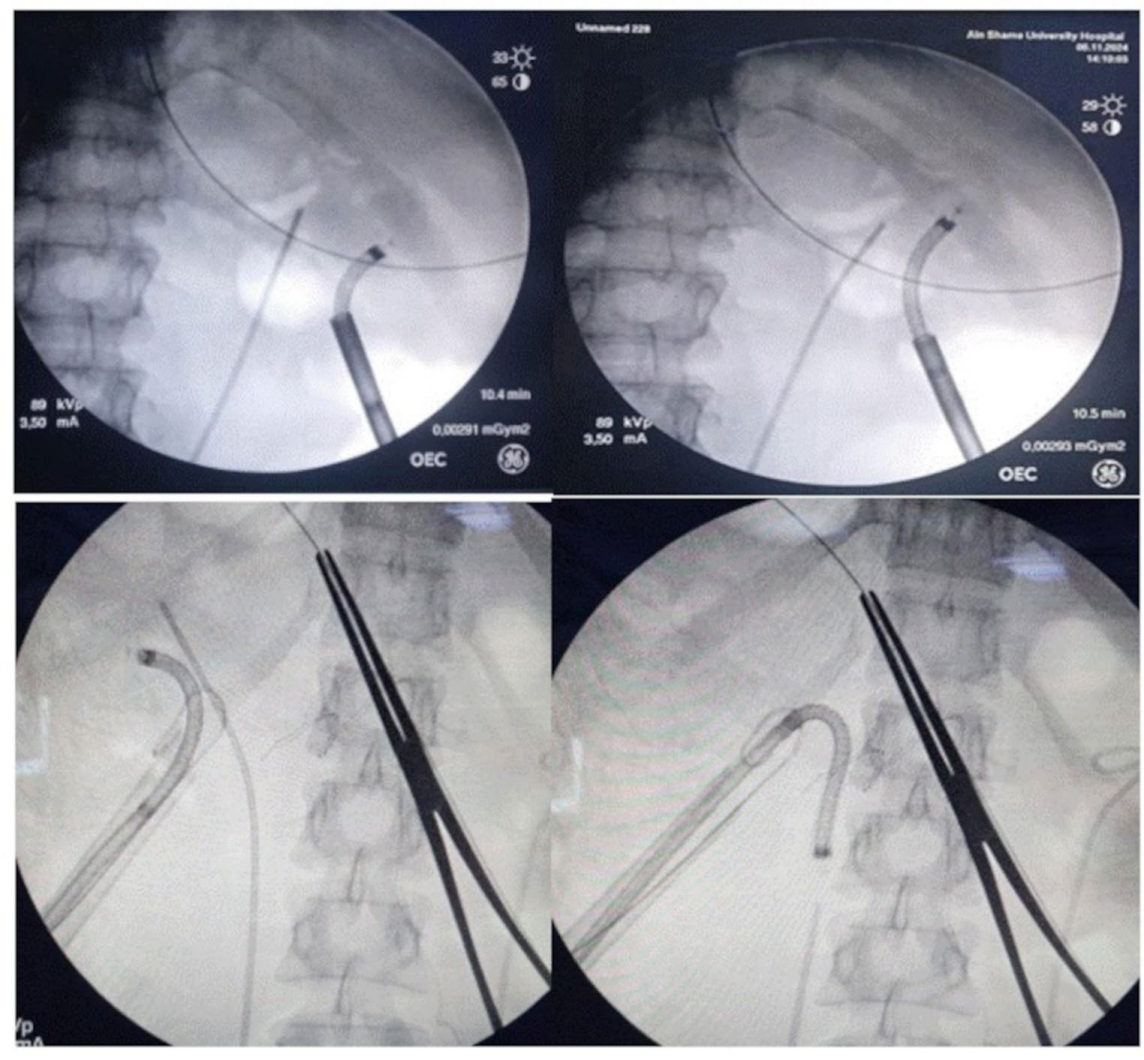
Intra-renal navigation of the cysto-nephroscope

This higher SFR is due to better extraction and drainage of stone fragments either actively by basketing or by passive drainage due to the dependency of the access sheath or the whirlpool of the injected saline from the sheath itself or the ureteric catheter. In addition, some studies reported that the infundibulo-pelvic angle may impact the SFR of RIRS [15]. This anatomical variation has no impact on the percutaneous access. Intraoperative navigation of the flexible cysto-nephroscope is shown in (Fig. 2).

Yilmazel et al., in their single-arm study evaluating UMPCNL using flexible endoscopy, showed comparable results of SFR of 96% [8]. Desai et al. evaluated UMPCNL in kidney stones and reported a SFR of 83% [14]. Liu et al., evaluated mPCNL using the rigid nephroscope and RIRS and reported SFR of (86.2%) and (61.4%) respectively [16]. A randomized controlled trial (RCT) was conducted by Datta et al. to evaluate UMPCNL and RIRS, reported a SFR of 100% and 73% respectively [17].

Significant residual stones were reported in 3 patients representing 4.8% of the F-mPCNL group and 14 patients representing 22.2% of the RIRS group. With the higher residual rate, more auxiliary procedures were needed to achieve stone clearance as shown in (Table 3). F-mPCNL showed a higher SFR in a single session with less need for reintervention and hospital re-entry.

The duration of the procedure is related mainly to the stone burden, especially with the RIRS. On the other hand, PCNL can address a higher stone burden in a shorter time [6]. In our study, the operative time was 47.60 ± 14.54 min in the F-mPCNL group compared to 59.30 ± 20.10 in the RIRS group with a statistically significant difference (P-value ˂0.001). This could be explained by the irrigation and drainage through percutaneous access, dependent drainage system, and the usage of stone fragmentation and busketing during the F-mPCNL rather than dusting in the RIRS.

Yilmazel et al. reported an operative duration of 45.6 ± 4.8 min. in the F-mPCNL technique which is less if compared to our study, but the mean stone size in their study was 1.53 ± 2.9 cm compared to the mean stone size of 2.31 ± 0.49 cm in the F-mPCNL group of our study [8]. A meta-analysis by Liu et al., reported shorter operative time for the mPCNL in comparison to RIRS [5].

F-mPCNL showed a fluoroscopy time of 46.14 ± 6.35 s. which represented the time for ureteric catheter application, percutaneous puncture, single-step dilatation with/without nephrostomy, and DJ stent antegrade insertion. This time was less in comparison to 68.51 ± 18.10 in the RIRS group which represented serial dilatation of the ureter, ureteral access sheath insertion, retrograde study, and DJ stent insertion at the end of the procedure. A study by Jain et al. reported a mean fluoroscopy time in mPCNL of 56.78 s compared to 40.2 s in RIRS [18]. This difference compared to our study could be explained by the single tract dilatation we adopted in the F-mPCNL rather than serial dilatation. Also, we did not place nephrostomy tube and DJ stent routinely which limits the use of fluoroscopy in our study.

In the F-mPCNL group, DJ stent was inserted in 9 patients (14.5%) out of 62 patients, unlike the RIRS groups where it was inserted routinely in all cases to allow passage of stone fragments smoothly. This led to a lower incidence of LUTS and less need for another hospital admission and eventually was reflected in a better QoL of life in the F-mPCNL group based on the SF-36 questionnaire [13], as shown in (Table 3).

Yilmazel et al., reported that no DJ stent was needed in all operated 52 patients. Datta et al., reported that only 1 patient required DJ stent insertion. This difference may be due to our higher mean stone size of included patients compared to Yilmazel and Datta et al. [8, 17].

According to the Clavien-Dindo classification, we did not report any complications in grades 3,4 or 5. Grade 1 complication was in the form of postoperative pain which required narcotic analgesics. 20 patients (32.2%) of the F-mPCNL group required additional analgesics compared to 10 patients (15.9%) in the RIRS group with a highly statistically significant difference. This could be attributed to the percutaneous puncture even though it was done with a medium-sized tract of 16.5 Fr.

Yilmaz et al. reported 2 (3%) cases of postoperative fever and 1 case represented a grade 3a complication and required DJ stent insertion for an obstructing ureteric stone [8]. Datta et al. reported that RIRS groups showed a higher incidence of postoperative fever and pain than UMPCNL groups [17].

We performed cost analysis in our study as shown in (Table 4). Although the hospital stay was significantly higher in the F-mPCNL compared to RIRS due to the longer hospital stay. The overall cost as well as the procedural cost was lower in the F-mPCNL.

Yilmazel et al. reported in their trial that UMPCNL was cost-effective based on their single-arm trial [8]. In the RCT by Datta et al., UMPCNL was more cost-effective than the RIRS with a highly statistically significant difference [17].

To our knowledge, our study is the first RCT to evaluate RIRS and F-mPCNL using a flexible cysto-nephroscope and mini-percutaneous access regarding efficacy, safety, QoL, retreatment rate and cost-effectiveness.

Our study’s limitations are that it was a single-centered study with a small study population. Also, we did not address the learning curve in each procedure. We recommend constructing a multicenter study with a larger study population.

## Conclusion

Both F-mPCNL and RIRS are safe and effective in treating renal stones. Compared to RIRS, F-mPCNL showed a better SFR in a single session, better QoL, less operative time, less radiational exposure, and less need for DJ stenting and postoperative auxiliary procedures. On the other hand, it was associated with more hospital stay and postoperative pain.

## Data Availability

Availability of data and materials: The datasets used and/or analyzed during the current study are available from the corresponding author upon reasonable request.

## Abbreviations

PCNL: Percutaneous nephrolithotomy
mPCNL: Mini-percutaneous nephrolithotomy
UMPCNL: Ultra-mini-percutaneous nephrolithotomy
F-mPCNL: Flexible percutaneous nephrolithotomy
RIRS: Retrograde intrarenal surgery
SFR: Stone free rate
QoL: Quality of life
NCCT: Non-contrast CT scan
ESWL: Extracorporeal shock wave lithotripsy
VA: Visual analog pain scale
USD: United States dollars
RCT: Randomized controlled trial

## Declarations

### Sample size calculation

Sample size was calculated using G-power 3.1.9.2 program, setting the type-1 error (α) at power at 80%. Results from a previous study (Abdulmecit Yavuz et al., 2020) showed that The SFR was 76% for the RIRS group compared to 94%; for the mini PCNL group (*p* < 0.001). Calculation according to these values produced a sample size of 61 cases in each group (122 total). However, the study will include 65 cases in each group (130 total) to account for dropout cases.

## References

[1] Kallidonis P et al (2020) Systematic review and Meta-Analysis comparing percutaneous nephrolithotomy, retrograde intrarenal surgery and shock wave lithotripsy for lower pole renal stones less than 2 cm in maximum diameter. J Urol 204(3):427–433. 10.1097/JU.0000000000001013 32150506 10.1097/JU.0000000000001013

[2] Kang SK, Cho KS, Kang DH, Jung H Do, Kwon JK, Lee JY (2017) Systematic review and meta-analysis to compare success rates of retrograde intrarenal surgery versus percutaneous nephrolithotomy for renal stones >2 cm, Medicine (Baltimore). 96(49):e911910.1097/MD.0000000000009119 10.1097/MD.0000000000009119 PMC5728962 29245347

[3] Mahmoud MA, Shawki AS, Abdallah HM, Mostafa D, Elawady H, Samir M (2022) Use of retrograde intrarenal surgery (RIRS) compared with mini-percutaneous nephrolithotomy (mini-PCNL) in pediatric kidney stones. World J Urol 40(12):3083–3089. 10.1007/s00345-022-04186-x 36244014 10.1007/s00345-022-04186-x PMC9712365

[4] Constantinou BT et al (2024) PCNL vs. two staged RIRS for kidney stones greater than 20 mm: systematic review, meta-analysis, and trial sequential analysis. Minerva Urol Nephrol 76(1). 10.23736/S2724-6051.23.05577-5 10.23736/S2724-6051.23.05577-5 38426420

[5] Liu Y et al. (2023) Efficacy and safety of minimally invasive percutaneous nephrolithotomy versus retrograde intrarenal surgery in the treatment of upper urinary tract stones (> 1 cm): a systematic review and meta-analysis of 18 randomized controlled trials, BMC Urol. 23(1)171 10.1186/s12894-023-01341-3 10.1186/s12894-023-01341-3 PMC10598962 37875837

[6] Türk C et al (2016) EAU guidelines on interventional treatment for urolithiasis. Eur Urol 69(3):475–482. 10.1016/j.eururo.2015.07.041 26344917 10.1016/j.eururo.2015.07.041

[7] Tawfeek AM, Arafa H, Higazy A, Radwan A, Tawfick A (2024) Is supine a preferred position for percutaneous nephrolithotomy in the pediatric age group? A randomized controlled study. Minerva Urol Nephrol 76(1). 10.23736/S2724-6051.23.05496-4 10.23736/S2724-6051.23.05496-4 38426422

[8] Yilmazel FK, Cinislioglu AE, Karabulut I, Yilmaz AH, Ozkaya F, Adanur S (2021) Ultra-mini flexible percutaneous nephrolithotomy in the treatment of moderate-size kidney stones: a new instrument, a preliminary prospective study. Urolithiasis 49(4):345–350. 10.1007/s00240-020-01225-3 33174122 10.1007/s00240-020-01225-3

[9] Jones P, Elmussareh M, Aboumarzouk OM, Mucksavage P, Somani BK (2018) Role of minimally invasive (Micro and Ultra-mini) PCNL for adult urinary stone disease in the modern era: evidence from a systematic review. Curr Urol Rep 19(4):27. 10.1007/s11934-018-0764-5 29516304 10.1007/s11934-018-0764-5 PMC5842282

[10] Kirecci S, Ilgi M, Yesildal C, Yavuzsan A, Albayrak A, Sarica K (2021) The impact of the pelvicalyceal anatomy characteristics on the prediction of flexible ureteroscopy outcomes. Urol Ann 13(2):105. 10.4103/UA.UA_19_20 34194134 10.4103/UA.UA_19_20 PMC8210722

[11] Ibarluzea G et al (2007) Supine Valdivia and modified lithotomy position for simultaneous anterograde and retrograde endourological access. BJU Int 100(1):233–236. 10.1111/j.1464-410X.2007.06960.x 17552975 10.1111/j.1464-410X.2007.06960.x

[12] Tawfeek AM, Elmoazen M, Saafan A, Higazy A, Radwan A, Gad HH (2021) Simultaneous antegrade and retrograde endourological approach in Galdakao-modified supine Valdivia position for the management of missed stents associated with complex renal stones: a non-randomized pilot study. Int Urol Nephrol 53(2):211–217. 10.1007/s11255-020-02639-z 32929666 10.1007/s11255-020-02639-z

[13] Donnally CJ et al (2011) Longitudinal evaluation of the SF-36 quality of life questionnaire in patients with kidney stones. Urol Res 39(2):141–146. 10.1007/s00240-010-0313-2 20924571 10.1007/s00240-010-0313-2

[14] Desai JD (2017) Ultra-mini PNL (UMP): Material, indications, technique, advantages and results. Arch Esp Urol 70(1):196–201. [Online]. Available: http://www.ncbi.nlm.nih.gov/pubmed/28221153 28221153

[15] Jessen JP, Honeck P, Knoll T, Wendt-Nordahl G (2014) Flexible ureterorenoscopy for lower pole stones: influence of the collecting system’s anatomy. J Endourol 28(2):146–151. 10.1089/end.2013.0401 24083332 10.1089/end.2013.0401

[16] Liu X et al (2022) Comparison of two techniques for the management of 2–3 cm lower pole renal calculi in obese patients. World J Urol 40(2):513–518. 10.1007/s00345-021-03872-6 34766214 10.1007/s00345-021-03872-6

[17] Datta SN, Chalokia RS, Patel KWWK, Janak RS (2022) Ultramini – percutaneous nephrolithotomy versus retrograde intrarenal surgery in the treatment of 10–30 mm calculi: a randomized controlled trial. Urolithiasis 50(3):361–367. 10.1007/s00240-022-01304-7 35107612 10.1007/s00240-022-01304-7

[18] Jain M, Manohar C, Nagabhushan M, Keshavamurthy R (2021) A comparative study of minimally invasive percutaneous nephrolithotomy and retrograde intrarenal surgery for solitary renal stone of 1–2 cm. Urol Ann 13(3):226. 10.4103/UA.UA_10_20 34421256 10.4103/UA.UA_10_20 PMC8343287

